# Plinabulin, a Distinct Microtubule-Targeting Chemotherapy, Promotes M1-Like Macrophage Polarization and Anti-tumor Immunity

**DOI:** 10.3389/fonc.2021.644608

**Published:** 2021-03-03

**Authors:** Marina Natoli, Petra Herzig, Elham Pishali Bejestani, Melanie Buchi, Reto Ritschard, G. Kenneth Lloyd, Ramon Mohanlal, James R. Tonra, Lan Huang, Viola Heinzelmann, Marta Trüb, Alfred Zippelius, Abhishek S. Kashyap

**Affiliations:** ^1^Department of Biomedicine, University Hospital Basel, University of Basel, Basel, Switzerland; ^2^Medical Oncology, University Hospital Basel, Basel, Switzerland; ^3^BeyondSpring Pharmaceuticals, New York, NY, United States

**Keywords:** immunoncology, macrophage polarization, anti-tumor immunity, ovarian cancer, chemotherapy

## Abstract

Reprogramming tumor infiltrating myeloid cells to elicit pro-inflammatory responses is an exciting therapeutic maneouver to improve anti-tumor responses. We recently demonstrated that a distinct microtubule-targeting drug, plinabulin—a clinical-stage novel agent—modulates dendritic cell maturation and enhances anti-tumor immunity. Here, we investigated the effects of plinabulin on macrophage polarization *in vitro* and *in vivo*. Plinabulin monotherapy induced significant tumor growth inhibition in mice bearing subcutaneous MC38 colon cancer. Importantly, the regressing tumors were characterized by an increase in M1-like/M2-like tumor-associated macrophages (TAM) ratio. The efficacy of plinabulin remained unaltered in T cell-deficient Rag2^−/−^ mice, suggesting an important role of macrophages in driving the drug's anti-tumor effect. Exposure of murine and healthy human macrophages to plinabulin induced polarization toward the M1 phenotype, including increased expression of co-stimulatory molecules CD80, CD86 and pro-inflammatory cytokines IL-1β, IL-6, and IL-12. M2-associated immunosuppressive cytokines IL-10 and IL-4 were reduced. This pro-inflammatory M1-like skewing of TAMs in response to plinabulin was dependent on the JNK pathway. Functionally, plinabulin-polarized human M1 macrophages directly killed HuT 78 tumor cells *in vitro*. Importantly, plinabulin induced a functional M1-like polarization of tumor infiltrating macrophages in murine tumors as well as in tumor samples from ovarian cancer patients, by preferentially triggering M1 proliferation. Our study uncovers a novel immunomodulatory effect of plinabulin in directly triggering M1 polarization and proliferation as well as promoting TAM anti-tumoral effector functions.

## Introduction

Tumor-associated macrophages (TAMs) are predictive of poor prognosis in the majority of advanced tumors and their presence is associated with increased tumor vascularization and resistance to chemotherapy (1). TAMs found in most tumors are predominantly of the M2 phenotype, which is associated with tumor-promoting functions and high expression of arginase-1, IL-10, CD163, and CD206 (2). Conversely, a higher infiltration of M1 TAMs—defined by high expression of IL-1β, inducible nitric oxide synthase (iNOS), CD80, CD86, and MHC class II molecules—correlates with good outcome in selected cancer types, including non-small cell lung cancer (NSCLC), hepatocellular carcinoma (HCC), ovarian and gastric cancers (3). M2 TAMs are directly suppressive to T cell responses, e.g., via upregulation of PD-L1 and release of IL-10, transforming growth factor-β (TGF-β) and reactive oxygen species (ROS)—as well as indirectly, via modulation of the tumor microenvironment, including recruitment of Tregs and inhibition of dendritic cells (DCs) (4).

Strategies aimed at depleting TAMs are therefore being explored for cancer immunotherapy, including targeting the CCL2-CCR2 (5, 6) and CXCL12–CXCR4 (7) axes to decrease recruitment of macrophages to the tumor sites, as well as targeting of CSF1R signaling (5, 8, 9) to increase TAM apoptosis. On the other hand, alternative therapeutic approaches are aimed at re-programming TAMs by promoting M1 pro-inflammatory and anti-tumoral functions. Agonistic targeting of CD40 leads to TAM-induced remodeling of the extracelluar matrix (ECM) thus increasing T cell infiltration (10). Combination of agonistic CD40 antibodies with dual VEGFA/Ang2 blockade was shown to enhance antitumor responses *in vivo*, in part by inducing proinflammatory (M1-like) macrophage activation (11).

It is well-documented that treatment with conventional chemotherapy, including anthracycline and taxane-based chemotherapy, provokes a number of immunomodulatory consequences, such as depletion of immunosuppressive subsets and induction of cancer immunogenic cell death and autophagy (12, 13). Microtubule destabilizing chemotherapy was shown to induce dendritic cell (DC) maturation—ultimately promoting anti-tumor T cell responses—via triggering the release of guanine nucleotide exchange factor-H1 (GEF-H1) in immature DCs and consequently activating Jun N-terminal kinase (JNK) (14). Here, we further investigated the immunomodulatory effects of microtubule destabilizing chemotherapy using plinabulin, a phase 3 clinical stage compound that has been assessed, among other indications, in patients with advanced solid tumors (NCT00322608), and is currently being advanced in a Phase 1/2 trial in combination with nivolumab in advanced lung cancer patients (NCT03575793) and in a phase 3 trial in combination with docetaxel in NSCLC (NCT02504489). In clinical studies, plinabulin has shown durable anti-cancer benefit in combination with docetaxel in NSCLC patients (15). Our findings uncover a novel and previously unreported effect of plinabulin in inducing polarization of tumor infiltrating macrophages to the M1-like phenotype both *in vitro* and *in vivo*. Importantly, plinabulin led to a functional M1-like polarization and preferential M1 proliferation, with enhanced secretion of pro-inflammatory M1-like cytokines and anti-tumoral functions.

## Materials and Methods

### Cell Lines

MC38 murine cell line was kindly provided by Thomas Wirth, Medizinischen Hochschule Hannover. EMT6 murine cell line and HuT 78 human cell line were purchased from the ATCC. The murine cell lines were cultured in DMEM medium containing L-glutamine (Sigma-Aldrich, D6429) and supplemented with 10% FCS (Pan Biotech, P30-5500), 1 × MEM non essential amino acids (Sigma-Aldrich, M7145), and 1 × penicillin/streptomycin (Sigma-Aldrich, P4333). The HuT 78 human cell line was maintained in RPMI medium containing L-glutamine (R8758, Sigma-Aldrich) supplemented with penicillin/streptomycin (100 ng/ml, Sigma-Aldrich) and 10% FBS (Sigma-Aldrich).

### Mice

C57BL/6N wild-type mice were obtained from the breeding facility of the Department of Biomedicine, University Hospital Basel or from Janvier Labs (France). Rag2^−/−^ C57BL/6N mice were bred in-house at the Department Biomedicine. All animals were bred and housed according to institutional guidelines and all experiments were performed in accordance with Swiss federal regulations (Basel Kantonal license numbers: 2370 and 2408). All experiments were carried out in mice between 8 and 16 weeks old and both males and females were used with no influence on results. All animals were maintained under a 12 h light cycle and given food and water available *ad libitum*.

### Primary Human PBMCs and Ovarian Cancer Samples

Human peripheral blood mononuclear cells (PBMCs) were isolated by density gradient centrifugation, using Histopaque (Sigma-Aldrich, 10771), from buffy coats obtained from healthy blood donors (Blood Bank, University Hospital Basel, Switzerland). Fresh tumor samples were obtained from two ovarian cancer patients undergoing tumor resections at University Hospital Basel, Switzerland. Patient characteristics are summarized in Supplementary Table 1. The study was approved by the local Ethical Review Board (Ethikkommission Nordwestschweiz) and University Hospital Basel, Switzerland. Written consent to use their tumor samples for research purposes was obtained from all patients. Fresh tumor samples were mechanically dissociated and digested using accutase (Innovative Cell Technologies, AT-104), collagenase IV (Worthington, LS004188), hyaluronidase (Sigma-Aldrich, H6254), and DNAse type IV (Sigma-Aldrich, D5025), directly after excision. Single-cell suspensions were prepared and samples were stored in liquid nitrogen until further use. In the following assays, single-cell suspensions derived from ovarian cancer samples were maintained in RPMI medium containing L-glutamine (Sigma-Aldrich, R8758) supplemented with 1 × penicillin/streptomycin (Sigma-Aldrich, P4333) and 10% FBS (Pan Biotech, P30-5500).

### *In vivo* Tumor Challenge and Treatment Protocol

C57BL/6N WT or C57BL/6N Rag2^−/−^ mice were injected subcutaneously into the right flank with 500,000 MC38 cells suspended in phenol red-free DMEM (without additives). After 18 days (named day 0), mice bearing established MC38 tumors (tumors ranging between 40 and 80 mm^3^) received peri tumoural injections of plinabulin (7.0 mg/kg, Beyondspring), vehicle (DMSO) or were left untreated on days 0, 1, 2, 4, 7, 9, and 11. Tumor volume was calculated according to the formula: D/2*d*d, with D and d being the longest and shortest tumor diameter in mm, respectively. In some experiments, mice were sacrificed on day 7, after injections of 7.5 mg/kg plinabulin (two doses per day, three times per week), the tumors were harvested and analyzed by flow cytometry.

### *In vitro* Differentiation and Treatment of Murine and Human Macrophages

For murine macrophage differentiation, bone-marrow cells were isolated from C57BL/6N WT mice and differentiated into macrophages by culturing the cells in complete RPMI supplemented with 20 ng/mL murine macrophage colony stimulating factor (M-CSF, Peprotech, 315-02) at 37°C for 7 days. On day 4 of the culture additional 4 mL of RPMI complete medium, supplemented with MCSF (20 ng/ml final) was added. On day 7, cells were washed with cold PBS and incubated with PBS containing EDTA (2 mM, Sigma-Aldrich). One hundred thousand cells were then cultured in a 96 well plate in complete RPMI supplemented with 20 ng/mL M-CSF at 37°C overnight. Next, cells were treated with plinabulin (Beyondspring) for 48 or 72 h at different concentrations or vehicle (0.1% DMSO),

Alternatively, tumor infiltrating macrophages were isolated from established MC38 tumors by harvesting the tumor, mechanically and enzymatically digesting it as detailed below and FACS sorting for CD11b^+^ F4/80^+^ cells. TAMs were then treated with 200 or 1,000 nM plinabulin for 48 h *ex vivo*.

For human macrophage differentiation, CD14^+^ cells were isolated from healthy human-derived PBMCs by positive selection using a MACS separation kit (Miltenyi Biotec, 130-050-201)—following the manufacturer's instructions—and treated with 50 ng/ml of human M-CSF (Peprotech, 300-25) for 6 days. At day 3 of the culture, medium was replaced with fresh medium containing 50 ng/mL M-CSF. Cells were then counted, seeded at the density of 200,000 cells per well in a 96-well plate with fresh medium (with M-CSF) and rested overnight prior to plinabulin treatment. Cells were treated with varying doses of plinabulin (Beyondspring) in the presence or absence of JNK inhibitor SP600125 (20 or 40 μM dose, Selleckchem) at 37°C for 48 or 72 h.

Primary human macrophages were obtained from ovarian cancer samples by thawing the tumor single cell suspensions, obtained as described above, and FACS sorting for CD11b^+^CD14^+^ cells using a BD Fortessa. Cells were then counted and 200,000 cells per condition were seeded in 96-well plates in RPMI medium with 50 ng/ml M-CSF and treated with plinabulin or control treatments for 48 h at 37°C.

In all cases, as control conditions, murine or human macrophages were treated with a combination of LPS (InVivo Gen−20 ng/mL for murine and 10 ng/mL for human experiments) and IFNγ (50 ng/mL, Peptrotech) or IL-4 (20 ng/mL for murine and 25 ng/mL for human experiments; Peprotech), to induce either an M1-like or M2-like polarization, respectively. Medium in these cultures was also supplemented with M-CSF (20 ng/mL for murine cells and 50 ng/mL for human cells). Proliferation and cell viability of macrophages was assessed on BD Fortessa. Additionally, absolute cell counts were calculated by flow cytometry using Precision Count Beads (Biolegend, 424902) following the manufacturer's instructions.

### Co-culture of Human Macrophages With Tumor Cells

For co-culture experiments, HuT 78 lymphoma cells were pre-stained using CTV (ThermoFisher, C34557), following the manufacturer's instructions and seeded at a density of 5,000 cells per well of a 96-well plate. Differentiated human macrophages were then added to the wells of the 96-well plate containing HuT 78 cells, at a density of 25,000 or 50,000 cells per well. The cells were co-cultured for 48 or 72 h at 37°C. Proliferation and cell viability of tumor cells was assessed on BD Fortessa. Additionally, absolute cell counts were calculated by flow cytometry using Precision Count Beads (Biolegend, 424902) following the manufacturer's instructions.

### Analysis of mRNA Expression

Human macrophages were isolated and differentiated as described previously. Total RNA was extracted using QIAGEN RNeasy kit (74104) and converted into cDNA using the iScript cDNA synthesis kit (Bio-Rad, 1708890). Next, SsoAdvanced Universal SYBR Green supermix kit (Bio-Rad, 1725270) was used for real-time qPCR according to the manufacturer's specifications, using a Thermo Fisher ABI ViiA7 machine. GAPDH was used as housekeeping gene. The primers sequences are reported in Supplementary Table 2.

### Measurement of Cytokine Production

For measurement of cytokine release, human macrophages were obtained as described above and treated with either plinabulin (200 nM) or combination of LPS (10 ng/ml) and IFNγ (50 ng/ml). Following 24 or 48 h of treatment, the 96-well plates were centrifuged at 1,500 rpm for 5 min and the cell culture supernatant was collected. Cytokine release was measured using a bead-based multiplex kit on a human M1/M2 macrophage panel (Biolegend, 740509), following the manufacturer's instructions.

### Tumor Cell Isolation for Flow Cytometry

For analysis of immune populations isolated from MC38 tumors, the following protocol was used. Harvested MC38 tumors were cut into smaller fragments with razor blades and then placed in 2–4 mL digestion mix [containing accutase (PAA), collagenase IV (Worthington), hyaluronidase (Sigma), and DNAse type IV (Sigma)] at 37°C for 45 min with constant shaking. Tumor suspensions were then filtered via 70 μM nylon mesh and washed with PBS containing EDTA (5 mM). Cell suspensions were subjected to red blood cell (RBC) lysis by adding 1 mL of RBC lysis buffer for 1 min, followed by a wash with PBS containing 2 mM EDTA. As a final step, cell suspensions were filtered via 70 μM nylon mesh and either stored in −80°C until further analysis or used directly for flow cytometry staining.

### Flow Cytometry

Single cell suspensions were washed with PBS and stained with the fixable live/dead UV Zombie dye (BioLegend). Cells were then blocked with Fc receptor-blocking anti-CD16/32 antibody (clone 2.4G2; 1:100) or with a human Fc Receptor Binding Inhibitor (Invitrogen, 1:100) for 20 min at 4°C. Next, cells were stained for cell surface antigens using the fluorophore-conjugated anti-murine or anti-human antibodies listed in Supplementary Table 3 for 20 min at 4°C. For intranuclear staining, cells were permeabilized and fixed using Invitrogen Fixation/Perm diluent (00-5223-56). Washing and antibody incubations were performed in FACS buffer (PBS, 0.5 mM EDTA, 2% FCS, 10% NaN3). Cells were either fixed with IC fix buffer (eBioscience, 00-8222-49) for 20 min or directly acquired on LSR Fortessa or FACS Aria III (BD Bioscience).

## Results

### Plinabulin Treatment Leads to Shrinkage of MC38 Tumors and Intratumoral Accumulation of M1-Like TAMs

**Figure 1:**
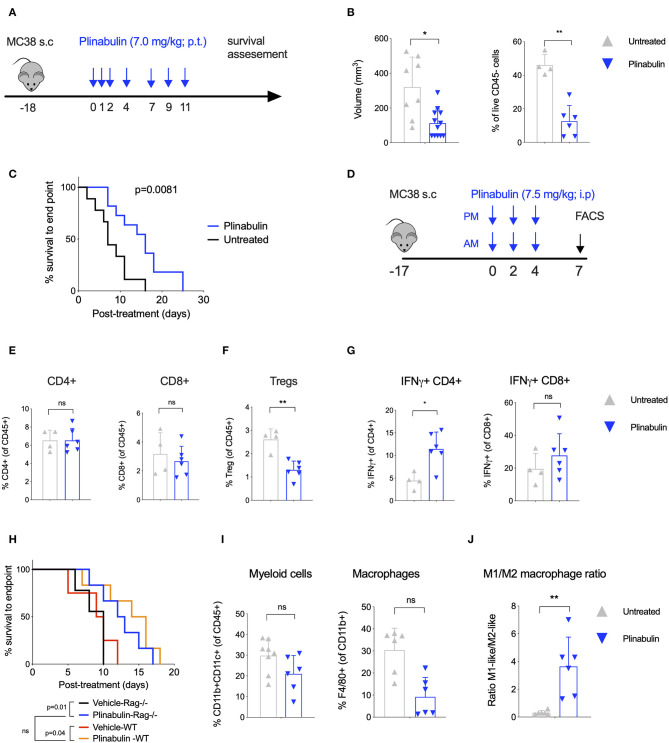
Treatment with plinabulin leads to tumor shrinkage and intratumoral accumulation of M1-like TAMs *in vivo*. **(A)** Mice bearing MC38 tumors (50–100 mm^3^) were left untreated or treated with plinabulin (7 mg/kg) administered peri-tumoral on days 0, 1, 2, 4, 7, 9, 11 post-treatment start (first dose administered 18 days post-MC38 cell innoculation). **(B)** Tumor volume (left) and percentage of live CD45^−^ cells out of total cells (i.e., tumor cells, right) 7 days post-plinabulin treatment start in plinabulin-treated or control mice. **(C)** Kaplan-Meier survival to humane endpoint curve of plinabulin-treated vs. control mice (log-rank test *p*-value is shown). **(D)** Mice bearing MC38 tumors (50–100 mm^3^) were left untreated or treated with plinabulin (7.5 mg/kg) administered intra-peritoneal twice a day on days 0, 2, 4, post-treatment start (first dose administered 18 days post-MC38 cell innoculation). Tumors were collected for flow cytometry analyses on day 7 post-treatment start. **(E)** Percentage of intratumoral CD4^+^ (left) and CD8^+^ (right) T cells out of total live CD45^+^ CD3^+^ cells in plinabulin-treated and untreated MC38 tumors. **(F)** Percentage of intratumoral Tregs in plinabulin treated and untreated MC38 tumors. **(G)** Percentage of IFNγ^+^CD4^+^ and IFNγ^+^ CD8^+^ cells after *ex vivo* anti-CD3 and anti-CD28 mAb re-stimulation of tumor infiltrating immune cells from plinabulin-treated or untreated MC38 tumors. **(H)** Kaplan-Meier survival curve of plinabulin-treated vs. untreated mice at the experiment endpoint in wild type C57BL/6 (WT) or T- and B-cell deficient C57BL/6 RAG^−/−^ mice. **(I)** Frequency of CD11b^+^ CD11c^+^ positive DCs (of all CD45^+^ cells, left) and F4/80^+^ Ly6C^−^ Ly6G^−^ TAMs (of CD11b^+^ population, right) in MC38-tumor bearing mice treated or untreated with plinabulin. **(J)** M1/M2 ratio (defined as the ratio between CD80^+^ and CD206^+^ TAMs) in plinabulin-treated MC38-tumor bearing mice and in untreated animals. Statistical significance was determined by Kolmogorov-Smirnov *t*-test **(B,E–G,I,J)** or log-rank Mantel-Cox test **(C,H)**. ns, not significant; *p < 0.05; **p < 0.01. Error bars show SD. Data are derived from two independent experiments with 4–6 animals in each group. Each symbol represents an animal.

We first investigated the anti-tumor activity of plinabulin *in vivo* in mice bearing subcutaneous MC38 colon cancer tumors. Mice with established tumors (50–100 mm^3^) were treated with seven doses of plinabulin (7.0 mg/kg; given peri-tumorally) spread over 11 days (Figure 1A), and tumor growth was measured. Plinabulin-treated mice had significantly smaller tumor volumes after 14 days of treatment compared to vehicle-treated mice (Figure 1B, left). Similarly, the percentage of live non-immune cells (CD45^−^ cells; tumor cells) was significantly reduced in the treated group (Figure 1B, right). Plinabulin-treated mice also had significantly prolonged survival (*p* = 0.0081, log-rank test) to the end point (Figure 1C). Similar findings were observed in the EMT6 breast cancer murine model (Supplementary Figures 1A,B).

Upon analysis of the tumor microenvironment by flow cytometry 7 days post-plinabulin treatment (as outlined in Figure 1D), no change in CD4^+^ (Figure 1E, left) and CD8^+^ T cell (Figure 1E, right) frequency was observed in plinabulin or control-treated mice. Plinabulin-treated MC38 tumors had significantly lower frequency of tumor infiltrating Tregs when compared to control tumors (Figure 1F). The tumor infiltrating T cells (both CD4^+^ and CD8^+^) of plinabulin-treated mice were functional, with increased capacity to produce intracellular IFN-γ, compared to control (Figure 1G), upon *ex vivo* re-stimulation with anti-CD3 and anti-CD28 monoclonal antibodies (mAbs). TNF-α expression remained unchanged in treated and control T cells (Supplementary Figure 1C). To assess the role of T cells in this system, MC38 tumor growth was monitored after plinabulin treatment in C57BL/6 Rag2^−/−^ mice, which lack T cells and B cells. Both Rag2^−/−^ and WT mice were equally sensitive to plinabulin, as tumor growth delay in response to plinabulin was observed in mice with and without T cells (Figure 1H). This suggests that T cells may not be the primary driver of efficacy to plinabulin in the MC38 tumor model.

Of note, we observed an overall reduction in CD11b^+^CD11c^+^ myeloid cells (Figure 1I, left) and particularly of F4/80^+^ TAMs (Figure 1I, right) in plinabulin-treated MC38 tumors. We also noted a significant increase in the M1-like to M2-like TAM ratio (defined as the ratio between CD80^+^ and CD206^+^ TAMs, Figure 1J), indicating an increased presence of anti-tumor M1-like TAMs. We subsequently characterized the effects of plinabulin on macrophage polarization and function.

### Plinabulin Induces M1 Polarization of Murine and Human Macrophages

**Figure 2:**
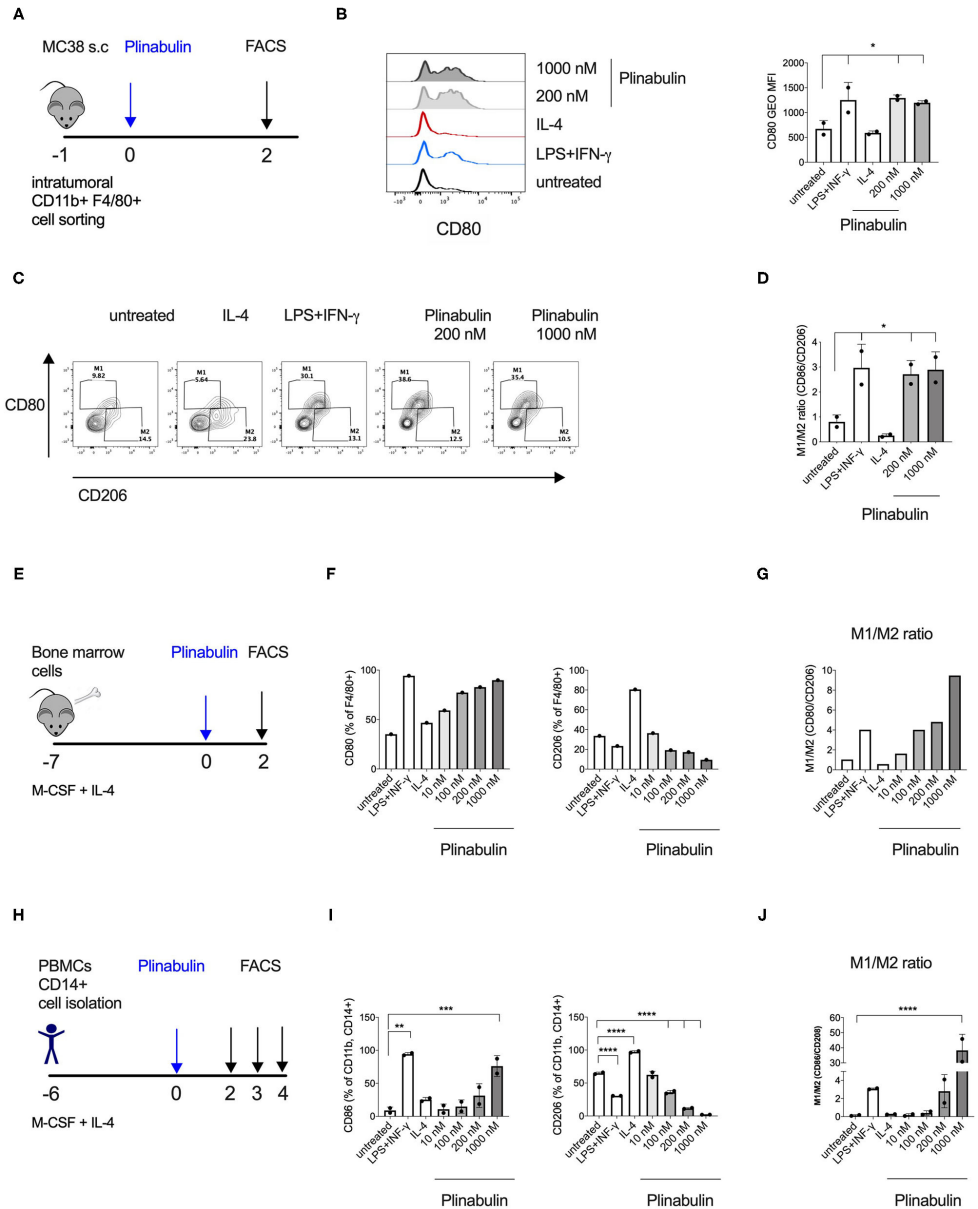
Plinabulin directly induces M1 polarization of murine and human macrophages *in vitro*. **(A)** MC38 tumors (400–600 mm^3^) from C57BL/6 mice were isolated (day −1). Tumor infiltrating TAMs were sorted by FACS and treated with plinabulin or relevant controls (day 0). Macrophage polarization was assessed by flow cytometry (day 2). **(B)** Left: Histograms depicting expression of CD80 in murine TAMs treated *ex vivo* for 48 h with plinabulin (1,000 or 200 nM), IL-4 (25 ng/mL) or LPS (20 ng/mL), and IFN-γ (50 ng/mL) combination and in untreated control. Right: gMFI of CD80 expression of murine TAMs in different treatment conditions (as indicated) after 48 h. **(C)** Density plots of CD80 and CD206 expression in murine TAMs treated with plinabulin or in control conditions for 48 h. **(D)** Quantification of M1/M2 ratio in murine TAMs treated with plinabulin or in control conditions for 48 h. **(E)** Murine BMDMs were generated by culturing murine bone marrow cells with M-CSF (20 ng/mL) and IL-4 (25 ng/mL) for 7 days. BMDMs were treated with plinabulin or control conditions for 2 days prior to assessment with flow cytometry. **(F)** Frequency of CD80^+^ (left) or CD206^+^ (right) cells out of F4/80^+^ BMDMs, treated with plinabulin or control conditions. **(G)** Quantification of M1/M2 ratio in murine BMDMs treated with plinabulin or control conditions for 48 h. **(H)** Experimental outline of macrophage generation with M-CSF (50 ng/mL) and IL-4 (25 ng/mL) from healthy donor PBMCs and treatment with plinabulin at indicated doses or controls (LPS at 25 ng/mL and IFN-γ at 50 ng/mL). **(I)** Frequency of CD80^+^ (left) or CD206^+^ (right) cells out of CD11b^+^ CD14^+^ human macrophages, treated with plinabulin or control conditions. **(J)** Quantification of M1/M2 ratio in human CD14^+^ derived macrophages treated with plinabulin or control conditions for 48 h. **(B,D,I,J)** Statistical significance was determined by one-way Anova with multiple comparisons to control group (untreated cells) (*p < 0.05; **p < 0.01, ***p < 0.001, ****p < 0.0001). Only statistically significant comparisons are shown. Error bars show SD. **(B,D)** Data derived from single experiment with two animals, each symbol represent an animal. **(F,G)** Data derived from a single experiment with BMDM pooled from 4 animals. **(I,J)** Data derived from single experiment with two donors, each symbol represents a donor.

In order to elucidate the direct effect of plinabulin treatment on murine macrophages, we isolated CD11b^+^F4/80^+^ TAMs from established MC38 tumors by FACS and treated them *ex vivo* with plinabulin for 48 h (Figure 2A). Murine TAMs treated with a combination of LPS (20 ng/ml) and IFNγ (50 ng/ml) or with IL-4 (20 ng/ml) alone served as M1- or M2-TAM controls, respectively. As expected, treatment with LPS and IFNγ led to an increase in CD80 expression in TAMs, while a decreased CD80 expression was observed in the IL-4 condition (Figures 2B,C). Strikingly, treatment with either dose (200 or 1,000 nM) of plinabulin resulted in a significant increase in the M1/M2 ratio (M1 marker: CD80 and M2 marker: CD206), similar to the LPS/IFNγ treatment (Figures 2C,D). Importantly, plinabulin did not alter the viability of TAMs. Similar to the control conditions, more than 90% of all cells were found to be alive after plinabulin treatment (Supplementary Figure 2A). Next, we assessed the effects of plinabulin on bone marrow derived macrophages (BMDM, Figure 2E). Similar to TAMs, plinabulin treatment of BMDMs led to a significant, dose-dependent increase in M1 markers CD80 and CD86 (Figure 2F, left and Supplementary Figure 2B, respectively) and a concomitant decrease in expression of CD206 (Figure 2F, right). Accordingly, we observed a dose-dependent increase in the M1/M2 ratio upon plinabulin treatment, similar to LPS/IFN-γ M1 control (Figure 2G and Supplementary Figure 2C).

As a next step, we investigated healthy human monocyte-derived macrophages (Figure 2H). Following 48 h of treatment with increasing doses of plinabulin or with control treatments (10 ng/ml LPS and 50 ng/ml IFN-γ or 25 ng/ml IL-4—alone in the presence of M-CSF), the expression of human M1 marker CD80 and M2 markers CD206 and CD163 were assessed by flow cytometry. Similar to murine BMDMs, treatment of human macrophages with plinabulin resulted in a dose-dependent increase in expression of CD80 (Figure 2I, left), a decrease of the M2 marker expression (Figure 2I, right and Supplementary Figure 2D) and a significant increase in the M1/M2 ratio (Figure 2J). This phenotype was sustained also at 72 and 96 h of culture (Supplementary Figures 2E,F).

### Plinabulin Induces M1-Macrophage Proliferation and Pro-inflammatory Cytokine Release

**Figure 3:**
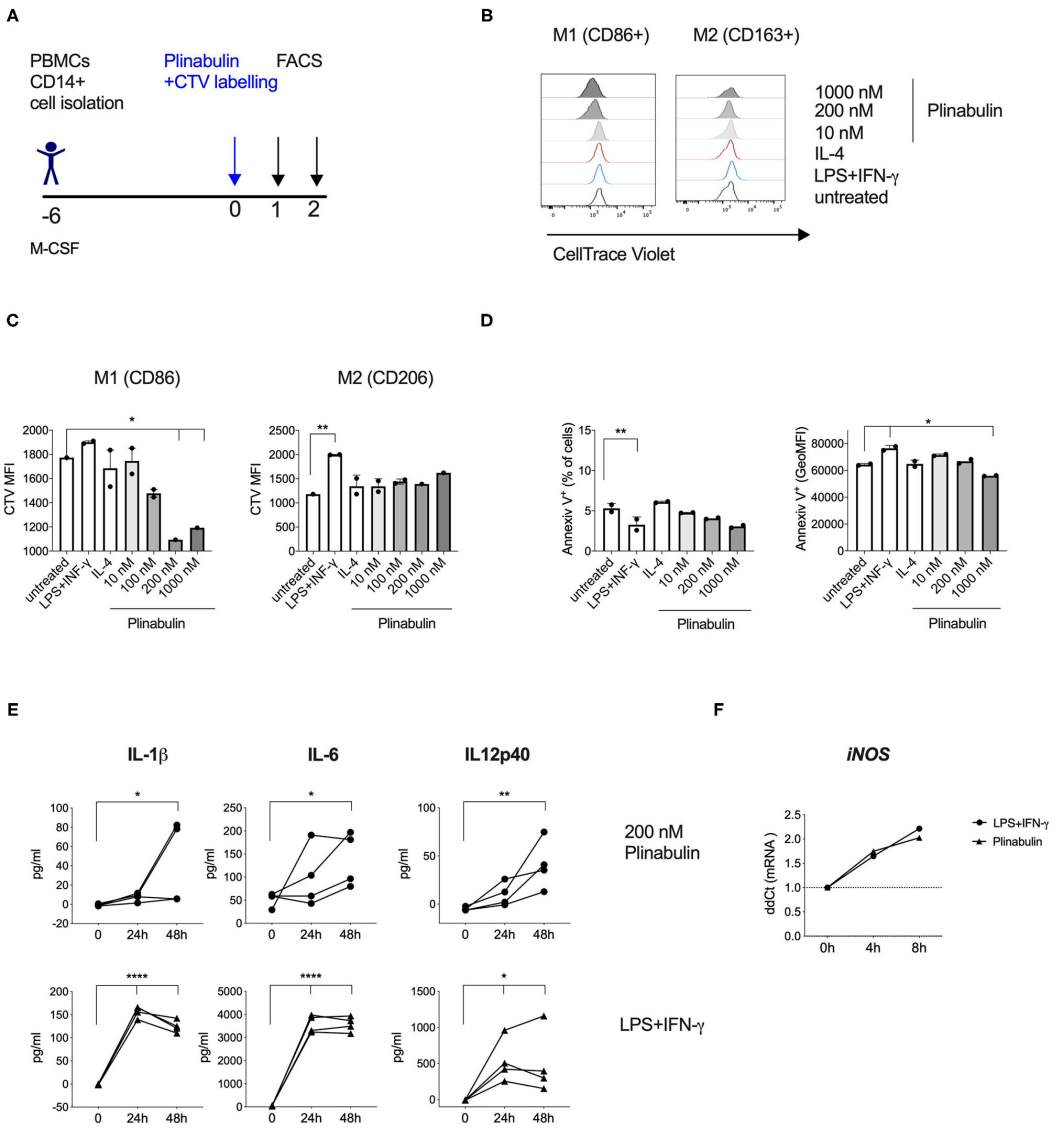
Plinabulin induces M1-macrophage proliferation and pro-inflammatory cytokine release. **(A)** Experimental outline of macrophage generation from healthy donor PBMCs, CTV labeling and treatment with plinabulin or controls prior to analysis by flow cytometry and multiplex cytokine analysis. **(B)** Histograms showing CTV expression, i.e., proliferation of CD86^+^ (left) or CD206^+^ (right) human macrophages treated for 48 h with plinabulin (1,000 or 200 nM), IL-4 (25 ng/mL), LPS (25 ng/mL), and IFN-γ (50 ng/mL), combination or untreated. **(C)** Quantification of CTV signal as gMFI in CD86^+^ (left) or CD206^+^ (right) human macrophages treated for 48 h with plinabulin or control treatments. **(D)** Percentage of AnnexinV^+^ cells (left) and gMFI of AnnexinV (right) in human macrophages treated for 48 h with plinabulin or control conditions. **(E)** Quantification of pro-inflammatory cytokinesIL-1β IL-6 and IL12p40 in the supernatant of human macrophages from four healthy donors treated for 0, 24, or 48 h with plinabulin (top) or LPS (25 ng/mL), and IFN-γ (50 ng/mL), combination treatment (bottom). **(F)** Quantification of iNOS mRNA expression by qPCR in human macrophages after 4 or 8 h of treatment with plinabulin or LPS (25 ng/mL), and IFN-γ (50 ng/mL), combination. **(C–F)** Statistical significance was determined by one-way Anova with multiple comparisons to control group (untreated cells for **C,D**; 0 h for **E,F**). *P*-values indicated on the graphs: *p < 0.05, **p < 0.01, ****p < 0.0001. Error bars show SD. **(C,D)** Data are derived from two independent experiments with samples pooled from two donors. Each symbol represents a pooled sample.

We next assessed whether the plinabulin-induced shift toward the M1 phenotype was accompanied by changes in macrophage proliferation and effector functions. Macrophages were differentiated from healthy human CD14^+^ monocytes (Figure 3A). Proliferation was measured as a dilution of the cell tracking violet (CTV) dye in the treated macrophages. Plinabulin treatment resulted in an increase in the proliferation of CD86^+^ macrophages (M1) as indicated in the histograms in Figure 3B (left). Interestingly, no such proliferation in CD86^+^ macrophages was observed in untreated and the M1 control (LPS/IFN-γ) and IL-4-treated groups. CD163^+^ macrophage (M2) proliferation remained unaltered in all conditions (Figure 3B, right). Dilution of the CTV was also quantified as mean fluorescence intensity (MFI, Figure 3C). We then performed staining with AnnexinV to analyze potential cell death induction in the different subsets. There were no significant changes in AnnexinV^+^ cells with plinabulin treatment when the total macrophage population or M1 and M2 subsets were analyzed (Figure 3D). These findings indicate that plinabulin-induced microtubule destabilization specifically and preferentially triggers M1-like macrophage proliferation, without inducing M2-like macrophage cell death.

Next, we assessed protein and gene expression of M1- or M2-specific cytokines. Macrophages treated with plinabulin or the LPS/IFN-γ control over 24 or 48 h showed increased capacity to produce M1-associated cytokines IL-1β, IL-6, and IL-12p40 (Figure 3E). Additionally, we detected increased gene expression of *iNOS* as early as 4 h after plinabulin treatment (Figure 3F) and of *IL-1*β as early as 6 h (Supplementary Figure 3A). Conversely, mRNAs for M2-associated genes *Egr2, Tgfb1, Il4*, and *Ccl17* remained downregulated up to 20 h post-plinabulin treatment (Supplementary Figure 3B).

### JNK Pathway Is Involved in Plinabulin-Induced M1-Macrophage Polarization and Proliferation

**Figure 4:**
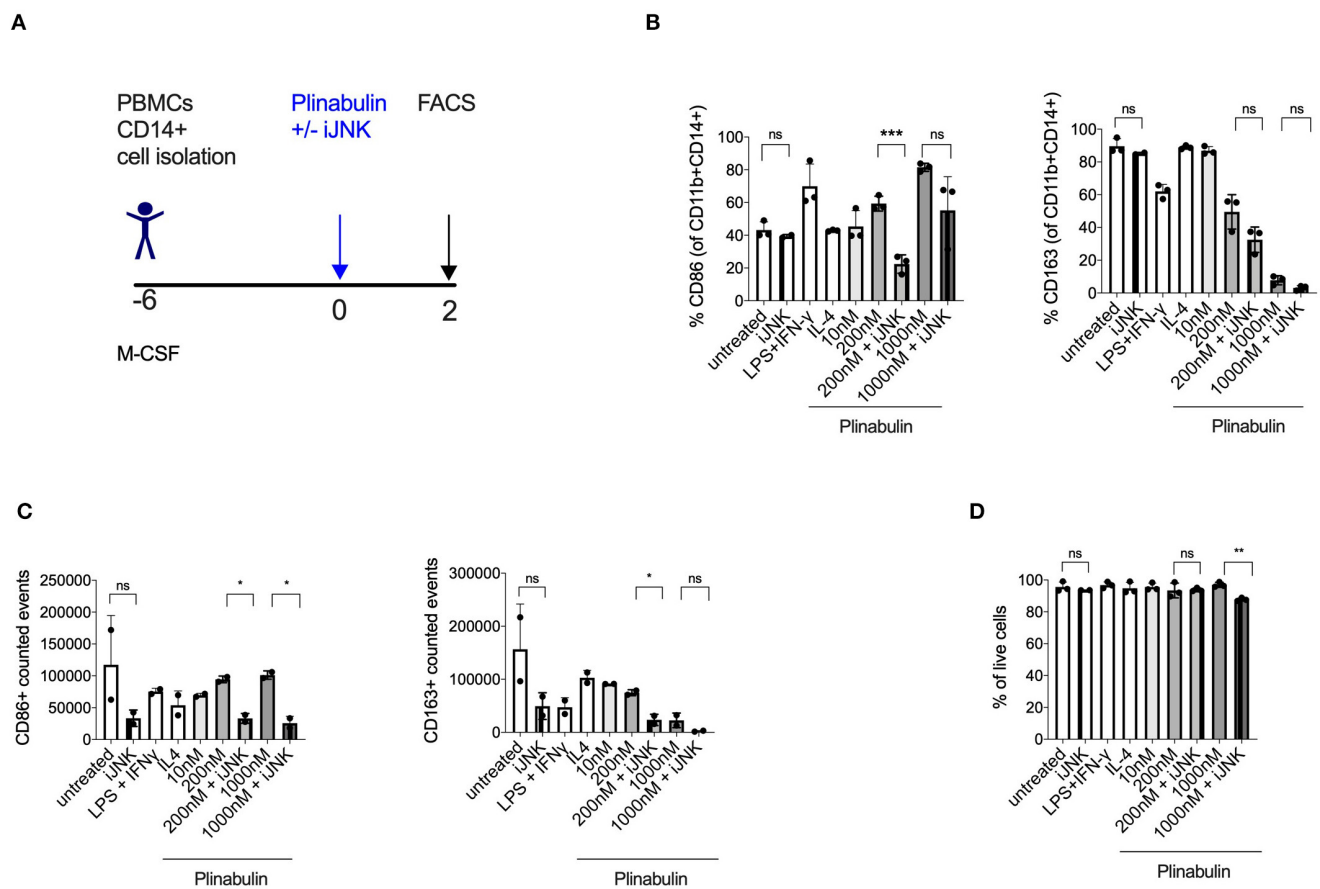
Plinabulin-induced M1 polarization is JNK-dependent. **(A)** Experimental outline of macrophage generation from healthy donor PBMCs and treatment with plinabulin at indicated doses or controls in the presence of a JNK inhibitor SP600125 (iJNK, 20 μM). **(B)** Percentage of CD86^+^ (left) or CD163^+^ (right) cells out of CD11b^+^ CD14^+^ human macrophages, treated with plinabulin or control conditions in the presence or absence of a JNK inhibitor. **(C)** CD86^+^ (left) or CD163^+^ (right) events out of CD11b^+^ CD14^+^ human macrophages, treated with plinabulin or control conditions in the presence or absence of a JNK inhibitor, calculated using counting beads on flow cytometry. **(D)** Percentage of live cells (cells negative for the live cell exclusion dye) out of total human macrophages, treated with plinabulin or control conditions, measured by flow cytometry. **(B–D)** Statistical significance was determined by Kolmogorov-Smirnov *t*-test between the indicated groups. *P*-values indicated on the graphs: ns, not significant; *p < 0.05; **p < 0.01, ***p < 0.001. Error bars show SD. Data are derived from two independent experiments: one with two individual donors and one with sample pooled from two donors.

Given our recent finding that microtubule destabilization triggers the activation of JNK in murine dendritic cells (14), we investigated the involvement of JNK in plinabulin-induced M1 polarization and proliferation of human macrophages *in vitro* (Figure 4A). Treatment with SP600125, a potent and specific JNK inhibitor, decreased CD86 expression in plinabulin-treated macrophages and thus resulted in partial reversion of the plinabulin effect on M1 polarization (Figure 4B, left). In contrast, JNK inhibition did not affect expression of CD163 (Figure 4B, right). Accordingly, upon assessing the total counts by FACS, M1 (CD86^+^) events were higher upon plinabulin treatment and this effect was partially lost upon JNK inihibiton; a similar effect was not observed on CD163^+^ events (Figure 4C). Of note, JNK inhibition did not significantly alter macrophage viability (Figure 4D). These findings suggest a role for plinabulin in directly inducing polarization of macrophages toward an M1-like phenotype and potentially the proliferation of these M1-like macrophages in a JNK-dependent fashion.

### Plinabulin-Polarized M1-Like Human Macrophages Induce Fas-Dependent Direct Killing of Tumor Cells

**Figure 5:**
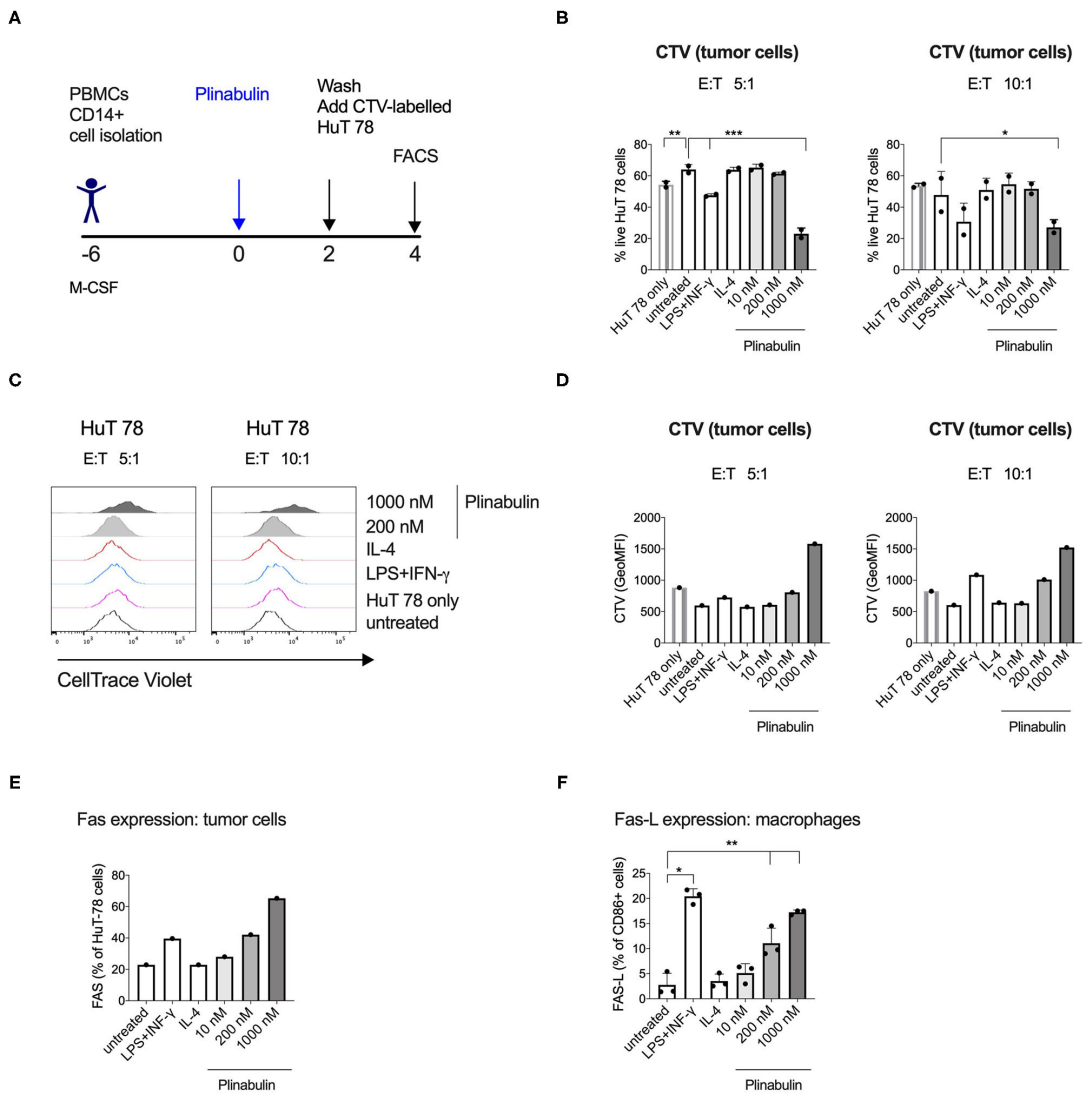
Human macrophages polarized with plinabulin have enhanced anti-tumoral effector functions. **(A)** Experimental outline of macrophage generation from healthy donor PBMCs, treatment with plinabulin or controls and co-culture with CTV-labeled HuT 78 tumor cells for 48 h. **(B)** Frequency of live HuT 78 tumor cells alone in culture or after co-culture with human macrophages that were pre-treated with plinabulin or control treatments at a 5:1 (left) or 10:1 (right) E:T ratio (E, i.e., effector= macrophages; T, i.e., tumor cells= HuT 78 tumor cells). **(C)** Histograms depicting CTV signal, i.e., proliferation of HuT 78 tumor cells after co-culture with human macrophages pre-treated with plinabulin or control treatments at a 5:1 (left) or 10:1 (right) E:T ratio. **(D)** Quantification of CTV signal as gMFI in HuT 78 tumor cells after co-culture with human macrophages pre-treated with plinabulin or control treatments at a 5:1 (left) or 10:1 (right) E:T ratio. **(E)** Frequency of Fas^+^ HuT 78 tumor cells after co-culture with human macrophages that were pre-treated with plinabulin or control conditions. **(F)** Frequency of Fas-L^+^ M1 macrophages (CD86+) treated with plinabulin or control conditions. **(B,E)** (Right): Statistical significance was determined by one-way Anova with multiple comparisons to control group (untreated cells). Only statistically significant comparisons are shown. *p < 0.05; **p < 0.01; ***p < 0.001. Error bars show SD. Data are derived from two independent experiments, each symbol represents an individual donor. **(D,E)** Data derived from a single experiment with one donor.

We next sought to determine the effector functions of plinabulin-polarized M1-like macrophages. CD14^+^ monocyte-derived macrophages were treated with plinabulin for 48 h. Upon removal of plinabulin by changing the culture medium, macrophages were co-cultured for further 48 h with HuT 78 tumor cells, pre-stained with CTV, at 5:1 and 10:1 macrophage to tumor cell ratios (Figure 5A). Tumor cell viability was measured by live/dead stain and tumor cell proliferation was determined by the dilution of the CTV dye.

Flow cytometry analysis showed a decrease in HuT 78 viability upon co-culture with 1,000 nM plinabulin-treated macrophages at either cell ratio compared to the untreated and the HuT 78 cells only controls (Figure 5B). A similar result in decreased tumor cell viability was seen in the LPS/IFN-γ M1 treatment control (Figure 5B). Similarly, HuT 78 proliferation was strongly inhibited when co-cultured with plinabulin (1,000 nM)-polarized human macrophages (Figures 5C,D), while there was a significantly increased proportion of live macrophages at this dose (Supplementary Figure 4).

Interestingly, we observed an increase in Fas^+^ tumor cells in co-culture with plinabulin-treated macrophages, compared to untreated cells and positive control (LPS/IFN-γ treatment, Figure 5E). Similarly, we observed a dose-dependent increase in Fas-L expression on human macrophages treated with plinabulin (Figure 5F). Taken together, our data demonstrate that plinabulin-polarized macrophages suppress tumor cell proliferation and increase tumor cell death, which is potentially mediated through Fas/Fas-L interaction.

### Plinabulin Triggers Functional Polarization of Ovarian Cancer Patient Tumor Infiltrating Macrophages

**Figure 6:**
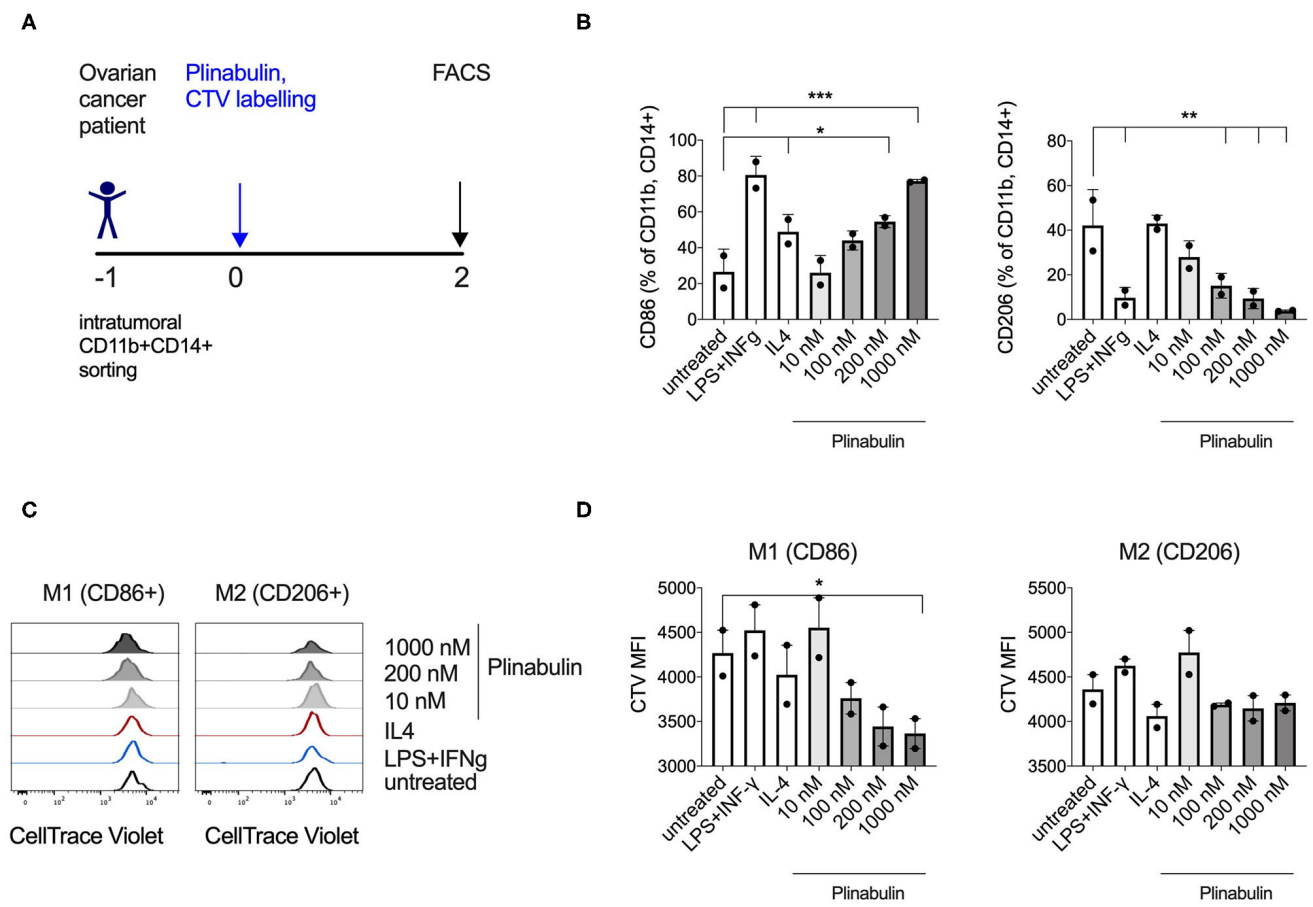
Plinabulin triggers polarization and proliferation of TAMs isolated from ovarian cancer patients. **(A)** Experimental outline of TAM isolation from ovarian patient tumor digests, CTV labeling and treatment with plinabulin or controls. **(B)** Frequency of CD86^+^ (left) or CD206^+^ (right) cells out of CD11b^+^ CD14^+^ human tumor infiltrating macrophages, treated with plinabulin or control conditions for 48 h. **(C)** Histograms showing CTV signal, i.e., proliferation of human ovarian TAMs treated *ex vivo* for 48 h with plinabulin (1,000 or 200 nM) or control treatments. **(D)** Quantification of CTV signal as gMFI in human ovarian TAMs treated *ex vivo* for 48 h with plinabulin (1,000 or 200 nM) or control treatments. **(B,D)** Statistical significance was determined by one-way Anova with multiple comparisons to control group (untreated cells). Only statistically significant comparisons are shown. *p < 0.05; **p < 0.01; ***p < 0.001. Error bars show SD. Data are derived from a single experiment. Each symbol represents a sample from one patient.

In order to investigate whether plinabulin induces M1 polarization of human TAMs, we FACS sorted CD11b^+^CD14^+^ cells from two tumor digests derived from ovarian cancer patients (predominantly of the M2 phenotype, Supplementary Figure 5), and treated them with plinabulin prior to staining with CTV (Figure 6A). Strikingly, after 48 h of treatment with plinabulin, a dose dependent increase in percentage of CD86^+^ TAMs was observed (Figure 6B, left). A similar increase was noted in the LPS/IFNγ M1 control treatment (Figure 6B, left). Conversely, expression of CD206 was significantly lower in plinabulin-treated cells compared to the untreated and IL-4 M2 control treatment (Figure 6B, right).

As with healthy human CD14^+^ derived macrophages, plinabulin treatment of intratumoral TAMs led to a preferential increase in the proliferation of M1 (CD86^+^) TAMs (measured by CTV dilution) as shown in the histograms in Figure 6C (left) and MFI quantified in Figure 6D (left), which was not observed for M2 TAMs (Figures 6C,D, right). Altogether, these data demonstrate that plinabulin treatment can re-polarize TAMs derived from cancer patients toward an M1-like phenotype as well as preferentially induce M1 proliferation.

## Discussion

The anti-tumor mode of action of microtubule-targeting agents (MTAs) is predominantly through mitotic spindle arrest and subsequent activation of apoptotic pathways and tumor cell death (13, 16, 17).

However, MTA plinabulin binds to the colchicine pocket of β-tubulin, in α,β-tubulin heterodimers, at a distinct site and with predicted kinetics that differ from colchicine and other tubulin-targeting agents (18). Plinabulin-bound tubulin heterodimers are prevented from polymerizing into microtubules (19), which ultimately affects cellular functions in a cell-type specific manner. In line with this, while plinabulin selectively decreases tumor blood flow by eliminating endothelial cells in tumor blood vessels (16, 20), it has also been shown to increase the maturation of dendritic cells (14) and to induce apoptosis of cancer cells (16). Potentially relevant to the ability of plinabulin to alleviate chemotherapy-induced neutropenia-, an indication for which plinabulin is in Phase 3 clinical testing (NCT03294577)—targeting tubulin with plinabulin boosted the number of hematopoietic stem/progenitor cells in the bone marrow of tumor bearing mice (21).

Nevertheless, the effects of plinabulin on the immune tumor microenvironment in tumor models and cancer patients remains relatively unexplored. This is particularly important as the prospect to enhance current cancer immunotherapies via rational combinations and by modulating distinct steps in the cancer-immunity cycle offers great clinical potential (22, 23).

In our study, we confirmed that plinabulin exhibits single agent efficacy *in vivo* in syngeneic MC38 and EMT6 murine tumor models, which are both myeloid-dominated (24–26) and therefore appropriate models to study myeloid cells. Indeed, multiple studies (25, 26) show that depletion of macrophages with an anti-CSFR1 antibody leads to lower tumor burden in the MC38 model, supporting the functional role of myeloid cells and particularly macrophages in altering tumor rejection.

Given the known role of plinabulin in inducing maturation and strong activation of DCs and subsequently enhancing anti-tumor immunity (14), we hypothesized that other myeloid cells are involved in the anti-tumor efficacy of plinabulin. Indeed, immunophenotyping revealed a macrophage-dominant phenotype, with increased proportion of M1-like TAMs in plinabulin-treated tumors. T cells were found to be non-critical in driving monotherapy efficacy of plinabulin, as suggested by our studies in T cell deficient Rag2^−/−^ animals. In fact, *in vitro* plinabulin treatment of TAMs isolated from MC38 tumors, murine BMDM and human monocyte-derived macrophages led to a dose-dependent increase in phenotypic and functional polarization toward the M1 phenotype. Of note, the dose of plinabulin used for our *in vitro* studies was demonstrated to be achievable in cancer patients treated with the drug (27). The plinabulin-induced M1 phenotype was characterized by upregulation of M1 surface markers as well as enhanced pro-inflammatory cytokine secretion, similar to classical M1-polarizing agents, such as LPS and IFN-γ (28, 29). However, plinabulin-induced M1-like polarization displayed unique characteristics not observed for classical *in vitro* polarized M1 macrophages, such as increased proliferation of M1-like macrophages as well as direct tumor killing upon exposure to plinabulin. This suggests that plinabulin may exhibit M1-like immunomodulatory properties which are mechanistically and functionally distinct from classic M1 polarization observed after LPS and IFN-γ stimulation *in vitro*. An unbiased approach, such as differential gene expression analysis, could perspectively help in determining the extent of overlap between classical and plinabulin-induced macrophage polarization.

Furthermore, our work shows that a crucial functional consequence of plinabulin treatment on human macrophages is their enhanced tumor cell killing capacity. Experimentally, we pretreated the macrophages with plinabulin and subsequently incubated them with tumor cells in the absence of the compound, to exclude any direct cytotoxic effects of plinabulin on tumor cells (16). We thus consider the observed increase in tumor cell death as a direct consequence of plinabulin-induced immunomodulation of human macrophages. Macrophages are capable of regulating self-apoptosis in an autocrine and paracrine way via the Fas-Fas-L axis. Additionally, macrophages are known to express both Fas and Fas-L and to upregulate and release soluble Fas-L upon activation by stimulation with immune complexes, PHA, or superantigen (30–32). In this work we hypothesize that the plinabulin-enhanced tumor cell killing by macrophages is at least partly dependent on the observed increased Fas-L expression on polarized TAMs. Additionally, we observed an increased Fas expression on Hu T78 tumor cells co-cultured with plinabulin-polarized macrophages. Fas-L deficiency in murine tumor models was shown to skew tumor-infiltrating myeloid cell populations toward an immunosuppressive phenotype and led to enhanced tumor burden (33). Another potential mechanism by which increased tumor cell death is observed in our study could be the increase in the secretion of pro-inflammatory cytokines, such as IL-1β, which was previously shown to induce tumor cell death in the presence of IFN-γ (34). It remains to be explored whether the enhanced tumor-killing capacity of plinabulin-polarized TAMs is exclusively contact-dependent and/or mediated by soluble factors, such as IL-1β.

Plinabulin is known to bind to tubulin in a differentiated manner (18), resulting in direct cytotoxic effects on tumor cells (16). It was therefore striking to find that in immune cells, namley macrophages and dendritic cells (14), plinabulin does not induce immune cell apoptosis and, on the contrary, enhances macrophage activation function and proliferation. Mechanistically, plinabulin-induced macrophage polarization and proliferation was shown to be largely dependent on JNK signaling.

Similarly, plinabulin-induced tumor cell apoptosis observed in previous studies was shown to be dependent on JNK signaling within tumor cells (16). Thus, treatment with plinabulin seems to present either pro- or anti-proliferative effects depending on the cell type, which may in turn depend on cell-specific JNK downstream signaling.

Similar to what we show here for macrophages, we have previously shown that the activation of the JNK pathway is critical for DC maturation in response to plinabulin treatment (14). In particular, GEF-H1 is released upon microtubule destabilization by plinabulin and is necessary for plinabulin-induced JNK pathway activation and subsequent DC activation (14).

A high-infiltration of TAMs correlates with poor prognosis in most solid tumors (35). In ovarian cancer, TAMs constitute the main population of immune cells within the tumor microenvironment (36). These cells are strongly implicated in the progression, metastasis and chemoresistance of ovarian cancer and are therefore a predictor of poor clinical outcome (37, 38). While platinum derivative chemotherapeutic compounds, such as cisplatin and carboplatin, may rather favor an increase in tumor-promoting M2 macrophages (39), we here show that exposure to plinabulin, a microtubule destabilizing drug, is able to re-polarize TAMs derived from ovarian cancer patients toward an M1-like phenotype and induce M1 proliferation. This suggests that plinabulin treatment is a viable therapeutic option for re-polarizing macrophages in human tumors.

Plinabulin therapy may be particularly attractive in combination with radiotherapy, which leads to increased influx of monocytes and conversion to M2 TAMs (40). Indeed, plinabulin application prior to radiotherapy was shown to increase treatment efficacy (41). Additionally, our findings provide a rationale to design combinations of plinabulin with immunotherapies targeting myeloid cells, in a synergistic effort to achieve enhanced anti-tumor immunity. In conclusion, our study supports the further development of plinabulin in clinical trials and offers key novel insights into the mechanism of action of MDAs in triggering TAM polarization toward an anti-tumoral phenotype.

## Data Availability Statement

The original contributions presented in the study are included in the article/Supplementary Material, further inquiries can be directed to the corresponding author/s.

## Ethics Statement

The studies involving human participants were reviewed and approved by the local Ethical Review Board (Ethikkommission Nordwestschweiz) and University Hospital Basel, Switzerland. The patients/participants provided their written informed consent to participate in this study. All animals were bred and housed according to institutional guidelines and all experiments were performed in accordance with Swiss federal regulations (Basel Kantonal license numbers: 2370 and 2408).

## Author Contributions

AK and AZ conceived the idea for the study. MN, PH, MT, AK, and AZ interpreted the data, made the figures, and wrote the manuscript draft. PH, EP, AK, GL, RM, JT, LH, and AZ planned the experiments and reviewed the manuscript draft. MN, PH, EP, MB, MT, and RR performed and analyzed the experiments. VH provided clinical samples. All authors reviewed and approved the manuscript.

## Conflict of Interest

AZ and AK received research funding from BeyondSpring. GL, RM, LH, and JT are employees of BeyondSpring and hold stock and/or stock options in BeyondSpring. The remaining authors declare that the research was conducted in the absence of any commercial or financial relationships that could be construed as a potential conflict of interest.

## Supplementary Material

Supplementary Figure 1 **(A)** EMT-6 tumor growth in untreated (black lines) or plinabulin-treated animals (blue lines; used at the dose of 15 mg/kg) over time. **(B)** Kaplan-Meier survival to humane end-point curve of EMT-6 tumor bearing, plinabulin-treated vs. untreated mice. Statistical significance was determined by log-rank Mantel-Cox test with *p*-value indicated on the graph. **(C)** Percentage of TNFα^+^CD4^+^ and TNFα^+^CD8^+^ cells after *ex vivo* anti-CD3 and anti-CD28 mAb re-stimulation of intratumoral CD4^+^ and CD8^+^ T cells from plinabulin-treated or untreated MC38 tumors.

Supplementary Figure 2 **(A)** Percentage live murine BMDMs following treatment with Plinabulin or controls as per schematic in Figure 2E. **(B)** Frequency of CD86^+^ cells out of F4/80^+^ BMDMs, treated with plinabulin or control treatments. **(C)** Quantification of M1/M2 ratio in BMDMs treated with plinabulin or control treatments. **(D)** Frequency of CD163^+^ cells out of CD11b^+^ CD14^+^ human macrophages, treated with plinabulin or control treatments. Statistical significance was determined by one-way Anova with multiple comparisons to control group (untreated cells). (^**^*p* < 0.01, ^****^*p* < 0.0001). Only statistically significant comparisons are shown. Error bars show SD. **(E)** Frequency of CD86^+^ cells out of CD11b^+^ CD14^+^ human macrophages after 48, 72, or 86 h of treatment with plinabulin or control treatments. **(F)** Frequency of CD206^+^ cells out of CD11b^+^ CD14^+^ human macrophages after 48, 72, or 86 h of treatment with plinabulin or control treatments.

Supplementary Figure 3 **(A)** Quantification of IL1β mRNA expression by qPCR in human macrophages after 6 or 18 h of treatment with plinabulin or LPS and IFN-γ combination. **(B)** Quantification of *Tgfb1, Egr2, Il4, and Ccl17* mRNA expression by qPCR in human macrophages after 4, 8, or 20 h of treatment with plinabulin or LPS and IFN-γ combination.

Supplementary Figure 4 Frequency of plinabulin or control-treated live human macrophages after co-culture with Fas^+^ HuT 78 tumor cells at 5:1 (left) or 10:1 (right) E:T ratio.

Supplementary Figure 5 Frequency of CD80^+^ and CD163^+^ cells in TAMs isolated from untreated human ovarian tumor digest.
