# Supplementary material for: Plinabulin, a Distinct Microtubule-Targeting Chemotherapy, Promotes M1-Like Macrophage Polarization and Anti-tumor Immunity
Source: Front Oncol. 2021 Mar 3;11:644608. doi: 10.3389/fonc.2021.644608 (PMC7966525; doi:10.3389/fonc.2021.644608)
Supplement: Supplementary file 1 [file Table_1.docx]

| **Patient ID** | **Cancer type** | **Histopathology** | **Stage** | **Treatment prior to surgery** | **Age (y)** |
| --- | --- | --- | --- | --- | --- |
| BS-620 | Epithelial ovarian cancer | High grade serous | IV | N | 71 |
| BS-823 | Epithelial ovarian cancer | High grade serous | IIIc | N | 54 |

**Table S1.** Patient characteristics
