# Supplementary material for: Plinabulin, a Distinct Microtubule-Targeting Chemotherapy, Promotes M1-Like Macrophage Polarization and Anti-tumor Immunity
Source: Front Oncol. 2021 Mar 3;11:644608. doi: 10.3389/fonc.2021.644608 (PMC7966525; doi:10.3389/fonc.2021.644608)
Supplement: Supplementary file 2 [file Table_2.docx]

| **Target gene** | **Forward primer** | **Reverse Primer** |
| --- | --- | --- |
| Ccl17 | GTGGTACCAGACATCTGAGGC | CTCTCTTGTTGTTGGGGTCCG |
| Egr2 | CAACATCTACCCGGTGGAGGAC | GATGCCTGCACTCACAATATTG |
| Gapdh | ACCCAGAAGACTGTGGATGG | TCTAGACGGCAGGTCAGGTC |
| Il10 | CTTCGAGATCTCCGAGATGCCTTC | ATTCTTCACCTGCTCCACGGCCTT |
| Il1β | GATAAGCCCACTCTACAGCTGG | GAAGACGGGCATGTTTTCTGCTTG |
| Il4 | CCGAGTTGACCGTAACAGACATC | GCACCCAGGCAGCGAGTGTC |
| Inos | AGGGACAAGCCTACCCCTC | CTCATCTCCCGTCAGTTGGT |
| Tgfb1 | CCCAGCATCTGCAAAGCTC | GTCAATGTACAGCTGCCGCA |

**Table S2.** Primers used for qPCR
