# Supplementary material for: Plinabulin, a Distinct Microtubule-Targeting Chemotherapy, Promotes M1-Like Macrophage Polarization and Anti-tumor Immunity
Source: Front Oncol. 2021 Mar 3;11:644608. doi: 10.3389/fonc.2021.644608 (PMC7966525; doi:10.3389/fonc.2021.644608)
Supplement: Supplementary file 3 [file Table_3.docx]

| **Fluorochrome** | **Target** | **Clone** | **Company** | **Cat. Number** | **Species reactivity** |
| --- | --- | --- | --- | --- | --- |
| PE | Annexin V | / | Biolegend | 640908 | Human/Mouse |
| FITC | CD11b | ICRF44 | BD | 562793 | Human |
| APC | CD14 | 63D3 | Biolegend | 77474 | Human |
| PerCP-eFluor 710 | CD14 | 63D3 | eBioscience | 9046-0149-120 | Human |
| PE-Cy7 | CD163 | GH1/61 | Biolegend | 333614 | Human |
| PE | CD206 | 15-2 | Biolegend | 321106 | Human |
| PE-CF594 | CD3 | UCHT1 | BD | 562280 | Human |
| PE-Cy7 | CD69 | GH1/61 | Biolegend | 333614 | Human |
| BV605 | CD80 | L307.4 | BD | 563315 | Human |
| BV711 | CD86 | IT2.2 | Biolegend | 305440 | Human |
| PE | Fas | DX2 | Biolegend | 305608 | Human |
| BV605 | Fas-L | NOK-1 | BD | 744099 | Human |
| APC | Ki67 | Ki-67 | Biolegend | 3505014 | Human |
| PE-Cy7 | CD11b | M1/70 | eBioscience | 25-0112-82 | Mouse |
| BV711 | CD206 | CO68C2 | Biolegend | 141727 | Mouse |
| PE | CD206 | C068C2 | Biolegend | 141706 | Mouse |
| APC-Cy7 | CD3e | 145-2C11 | BD Pharmingen | 561042 | Mouse |
| BUV496 | CD4 | GK1.5 | BD Pharmingen | 564667 | Mouse |
| PerCP-Cy5.5 | CD45 | 30-F11 | eBioscience | 45-0451-82 | Mouse |
| BV605 | CD80 | 16-10A | Biolegend | 104729 | Mouse |
| APC | CD86 | GL-1 | Biolegend | 105012 | Mouse |
| BV785 | CD8a | 53-6.7 | Biolegend | 100750 | Mouse |
| APC | F4/80 | BM8 | Invitrogen | MF48005 | Mouse |
| BV421 | F4/80 | BM8 | Biolegend | 123132 | Mouse |
| PE | Fas-L | MFL3 | eBioscience | 12-5911-81 | Mouse |
| FITC | FoxP3 | FJK-16s | Invitrogen | 11-5773-82 | Mouse |

**Table S3.** Antibodies used for flow cytometry
