# Supplementary figures and images for: Plinabulin, a Distinct Microtubule-Targeting Chemotherapy, Promotes M1-Like Macrophage Polarization and Anti-tumor Immunity
Source: Front Oncol. 2021 Mar 3;11:644608. doi: 10.3389/fonc.2021.644608 (PMC7966525; doi:10.3389/fonc.2021.644608)

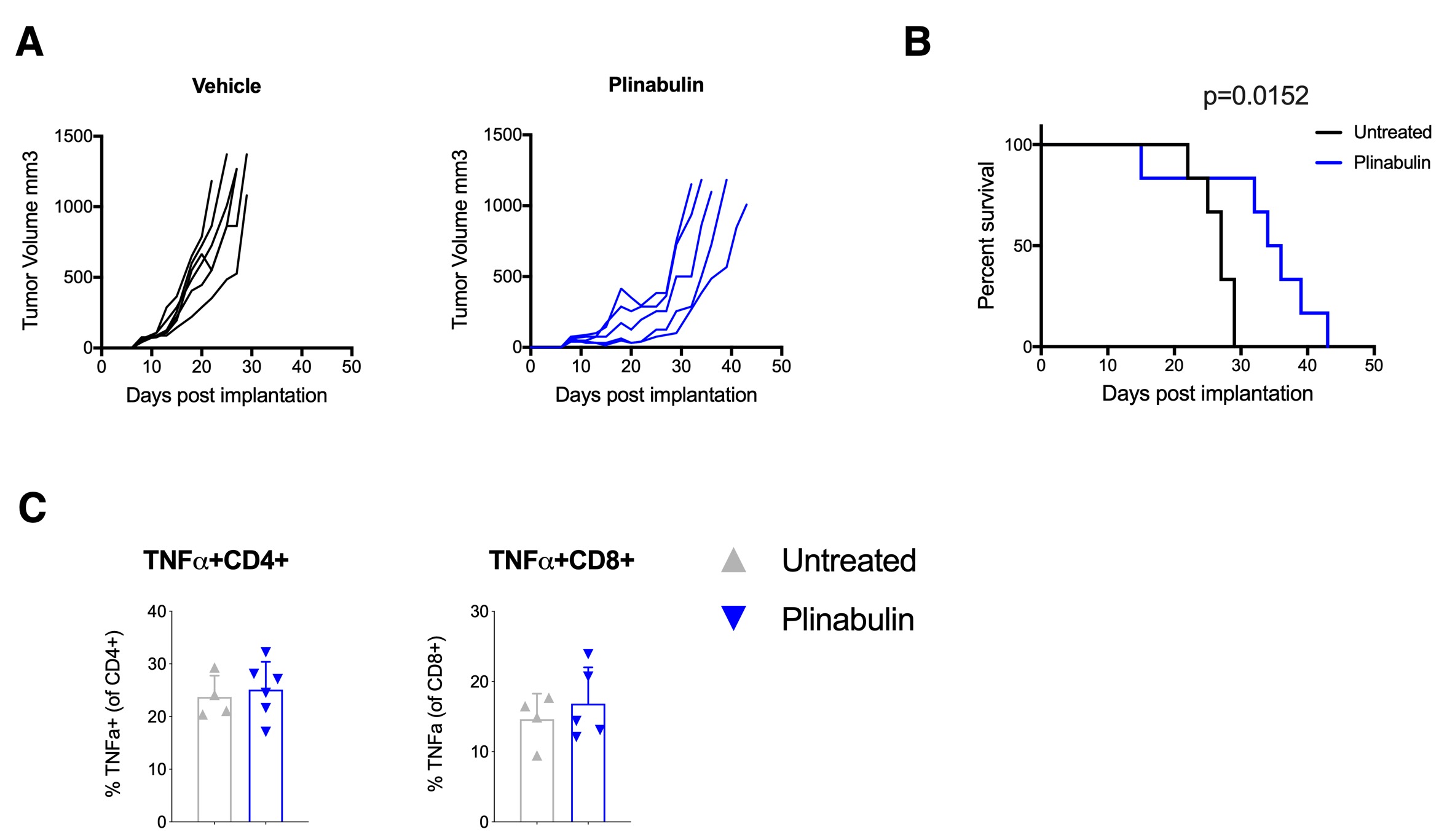

Supplement: Supplementary Figure 1 — (A) EMT-6 tumor growth in untreated (black lines) or plinabulin-treated animals (blue lines; used at the dose of 15 mg/kg) over time. (B) Kaplan-Meier survival to humane end-point curve of EMT-6 tumor bearing, plinabulin-treated vs. untreated mice. Statistical significance was determined by log-rank Mantel-Cox test with p-value indicated on the graph. (C) Percentage of TNFα+CD4+ and TNFα+CD8+ cells after ex vivo anti-CD3 and anti-CD28 mAb re-stimulation of intratumoral CD4+ and CD8+ T cells from plinabulin-treated or untreated MC38 tumors. [file Image_1.JPEG]

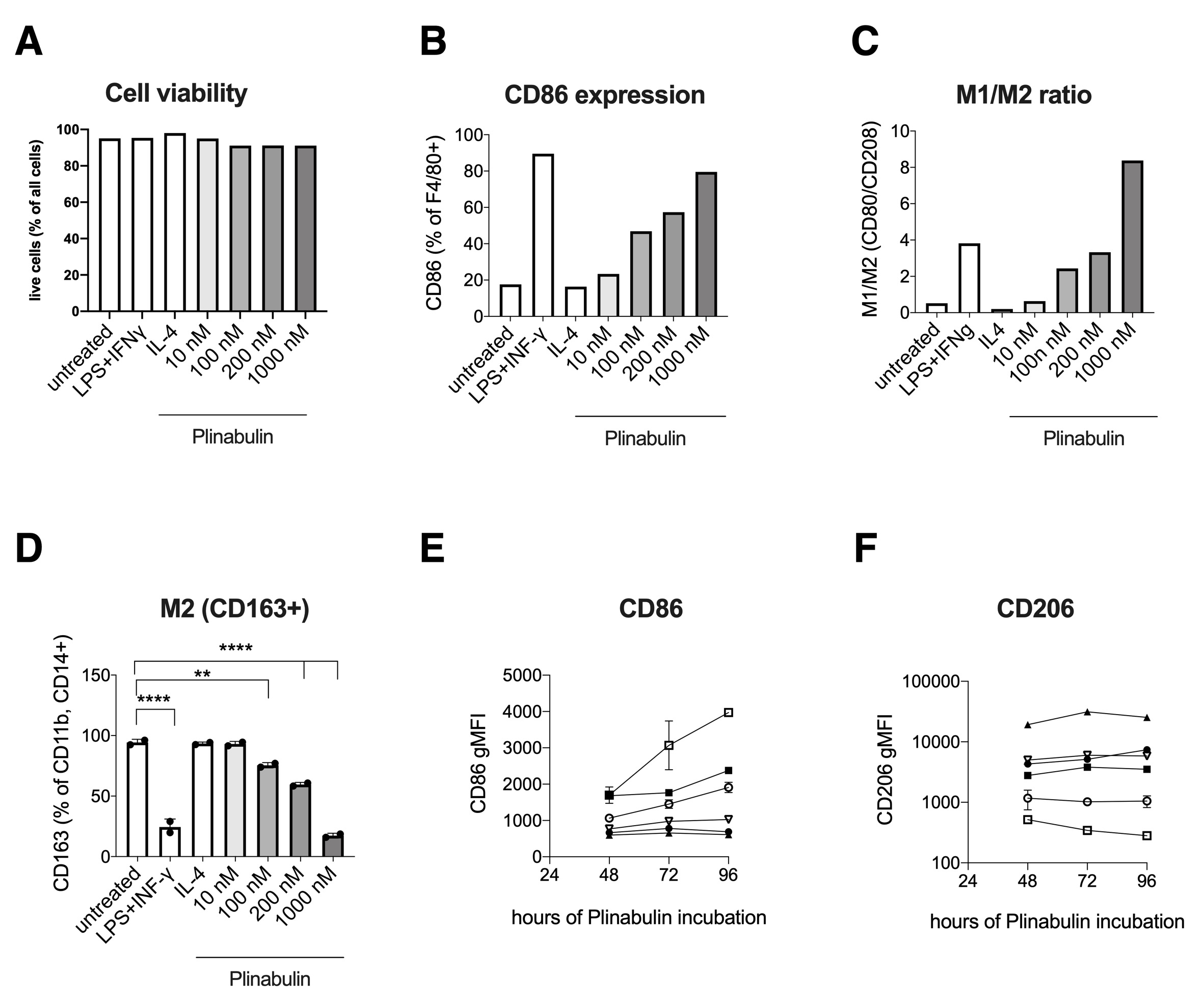

Supplement: Supplementary Figure 2 — (A) Percentage live murine BMDMs following treatment with Plinabulin or controls as per schematic in Figure 2E. (B) Frequency of CD86+ cells out of F4/80+ BMDMs, treated with plinabulin or control treatments. (C) Quantification of M1/M2 ratio in BMDMs treated with plinabulin or control treatments. (D) Frequency of CD163+ cells out of CD11b+ CD14+ human macrophages, treated with plinabulin or control treatments. Statistical significance was determined by one-way Anova with multiple comparisons to control group (untreated cells). (**p < 0.01, ****p < 0.0001). Only statistically significant comparisons are shown. Error bars show SD. (E) Frequency of CD86+ cells out of CD11b+ CD14+ human macrophages after 48, 72, or 86 h of treatment with plinabulin or control treatments. (F) Frequency of CD206+ cells out of CD11b+ CD14+ human macrophages after 48, 72, or 86 h of treatment with plinabulin or control treatments. [file Image_2.JPEG]

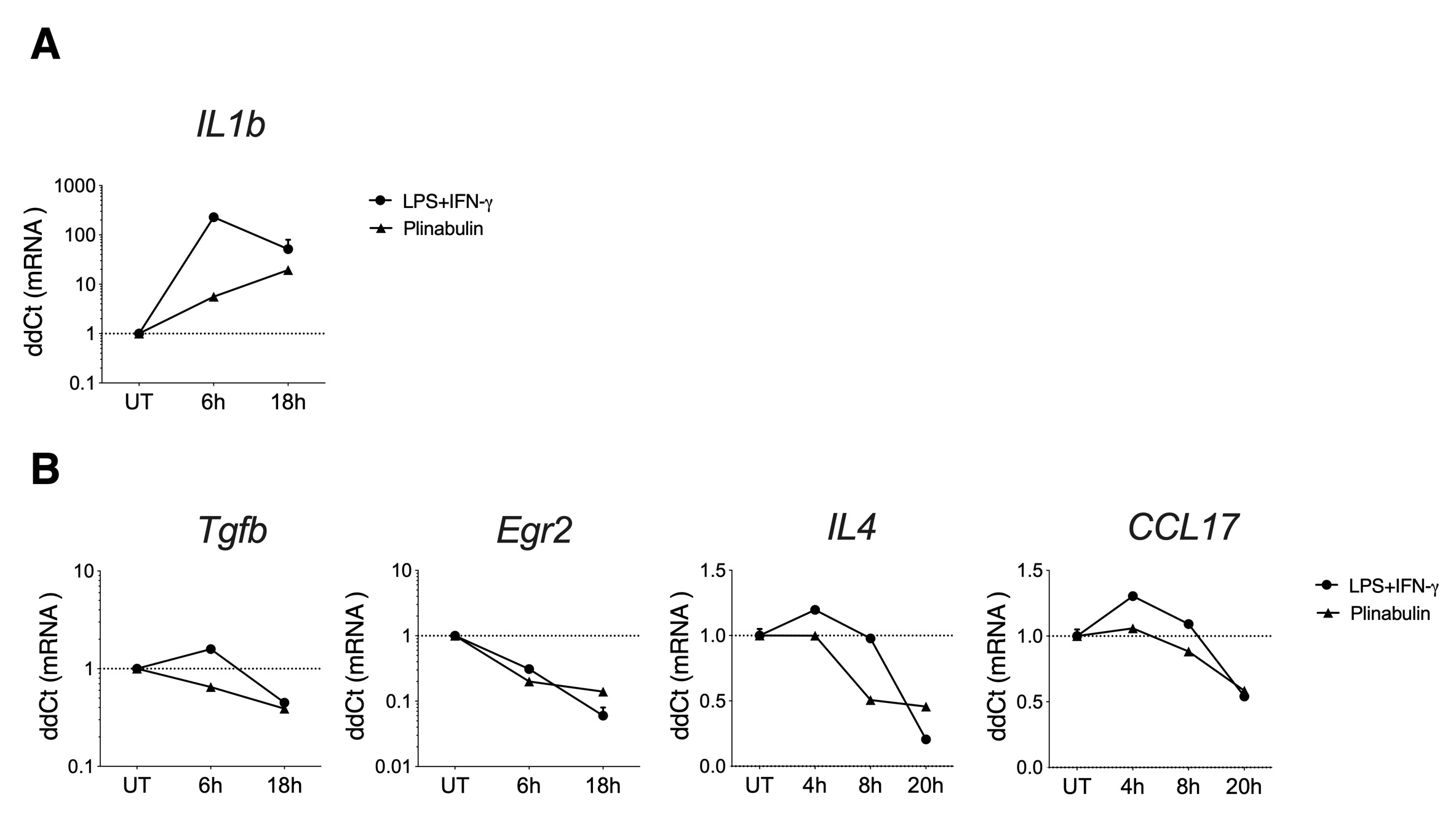

Supplement: Supplementary Figure 3 — (A) Quantification of IL1β mRNA expression by qPCR in human macrophages after 6 or 18 h of treatment with plinabulin or LPS and IFN-γ combination. (B) Quantification of Tgfb1, Egr2, Il4, and Ccl17 mRNA expression by qPCR in human macrophages after 4, 8, or 20 h of treatment with plinabulin or LPS and IFN-γ combination. [file Image_3.JPEG]

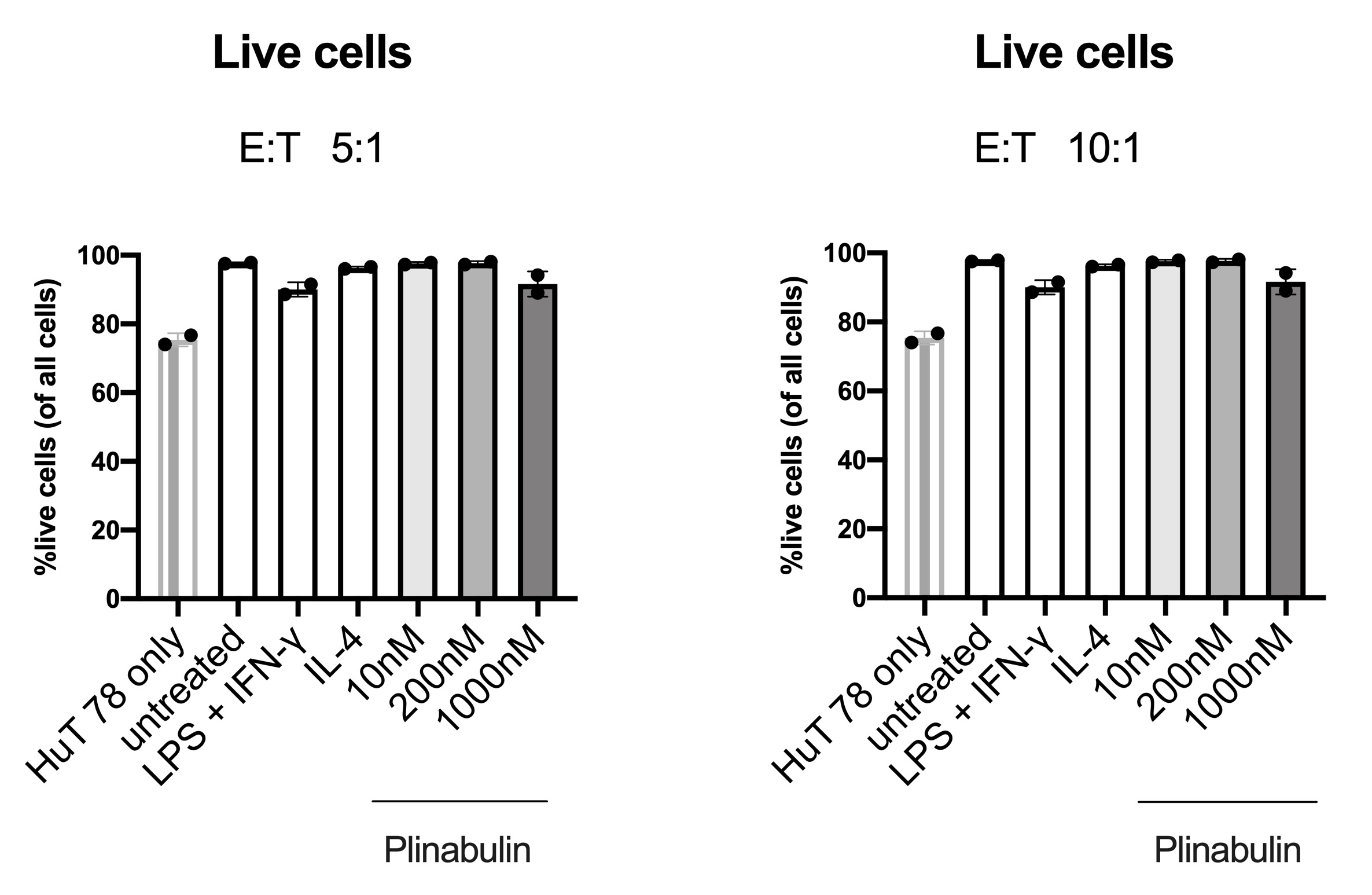

Supplement: Supplementary Figure 4 — Frequency of plinabulin or control-treated live human macrophages after co-culture with Fas+ HuT 78 tumor cells at 5:1 (left) or 10:1 (right) E:T ratio. [file Image_4.JPEG]

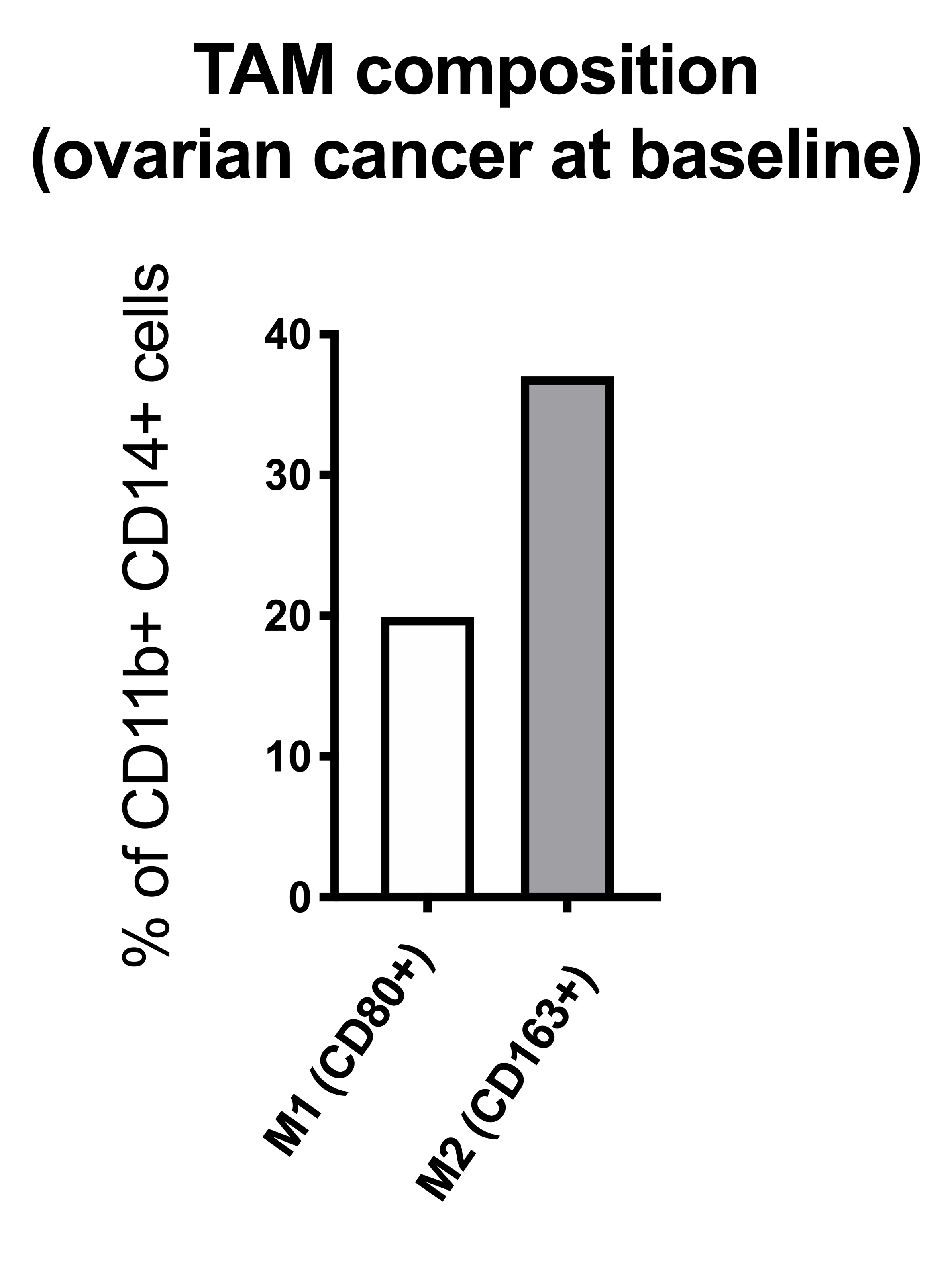

Supplement: Supplementary Figure 5 — Frequency of CD80+ and CD163+ cells in TAMs isolated from untreated human ovarian tumor digest. [file Image_5.JPEG]
